# Exercise-Induced Ventricular Tachycardia: A Case Report

**DOI:** 10.7759/cureus.47614

**Published:** 2023-10-24

**Authors:** Dibya S Mahanta, Anup K Budhia, Ranjan K Mohanty, Rama C Barik, Debasish Das, Debasis Acharya

**Affiliations:** 1 Cardiology, Institute of Medical Sciences and Sum Hospital, Bhubaneswar, IND; 2 Internal Medicine, Hi-Tech Medical College and Hospital, Bhubaneswar, IND; 3 Cardiology, Srirama Chandra Bhanja Medical College, Cuttack, IND; 4 Cardiology, All India Institute of Medical Sciences, Bhubaneswar, IND

**Keywords:** myocarditis, delayed after depolarisation, catecholaminergic polymorphic ventricular tachycardia, arrhythmogenic right ventricular cardiomyopathy, mitral valve prolapse, ventricular fibrillation

## Abstract

Exercise-induced ventricular tachycardia (EIVT) is a very rare condition that can occur in both structurally normal and abnormal hearts. It is important to recognize and understand the triggers, symptoms, and implications of this condition. In this report, we present a case of a young patient who experienced symptoms of palpitation, presyncope, and syncope during exertion. We also review the pathophysiology, clinical presentation, diagnosis, and management of exercise-induced ventricular arrhythmia. This information is particularly important in the context of sports medicine and cardiovascular health.

## Introduction

Exercise-induced ventricular tachycardia (EIVT) is very rare and can occur in structurally normal or abnormal hearts. It may be monomorphic, polymorphic, or bidirectional. It can be caused by various conditions, such as idiopathic right ventricular outflow tract ventricular tachycardia (RVOT VT), ventricular noncompaction, acute coronary syndrome, Brugada syndrome, arrhythmogenic right ventricular cardiomyopathy (ARVC), catecholaminergic polymorphic ventricular tachycardia (CPVT), Anderson-Tawil syndrome, mitral valve prolapse (MVP), digitalis toxicity, and myocarditis [1]. Catecholamine plays an important role in the initiation of tachycardia in RVOT VT, CPVT, ARVC, and also in acute coronary syndrome [1]. Serum catecholamine released during exercise increases intracellular cyclic adenosine monophosphate (cAMP), which leads to increased calcium ion overload within the cell. High intracellular calcium ions cause delayed after-depolarization or triggered activity, initiating tachycardia [2]. An increase in intracellular calcium ions also leads to changes in the duration of action potentials and the refractory period of cells. This can result in re-entry and increased automaticity, which can cause arrhythmia [2]. Catecholamine also has an inotropic, chronotropic, and dromotropic action on the myocardium, which further increases ischemia in ischemic myocardium. This increase in ischemia during exercise is the cause of EIVT in patients with coronary artery disease. We report a case about a 30-year-old female who presented with complaints of palpitations and syncope during physical activity. EIVT remains a topic of interest and concern for clinicians, sports medicine specialists, and athletes alike. Some types of EIVT can signal a more sinister underlying pathology or even pose a risk of sudden cardiac arrest. This case report and review of literature seeks to shed light on the intricacies of EIVT, exploring its mechanisms, implications, and contemporary approaches to its management.

## Case presentation

A married woman, aged 30, a homemaker by profession, visited us with palpitations, presyncope, and syncope symptoms during physical activities like climbing stairs, washing clothes, and walking briskly. She had no history of congenital or rheumatic heart disease, risk factors for coronary heart disease, or a family history of sudden cardiac death. During the physical examination, there were no notable findings in the cardiovascular system. The baseline ECG showed a normal sinus rhythm with a normal corrected QT interval. A structurally healthy heart was seen in the baseline echocardiogram. Routine blood tests, including the complete blood count (CBC), renal function test (RFT), liver function test (LFT), and thyroid function test, were also normal. The cardiac magnetic resonance imaging (MRI) did not detect any structural abnormalities and showed normal function of both ventricles.

**Figure 1:**
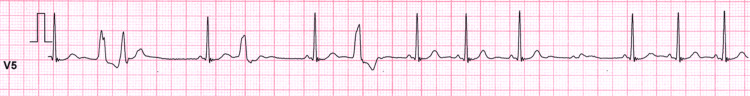
ECG at the start of the exercise test ECG at the start of the exercise test shows polymorphic ventricular premature complexes

**Figure 2:**
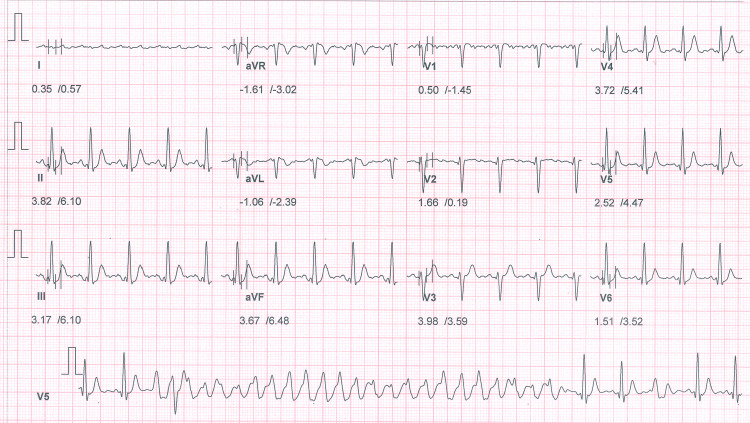
ECG just after stage one of the exercise test Polymorphic ventricular tachycardia induced during exercise

**Figure 3:**
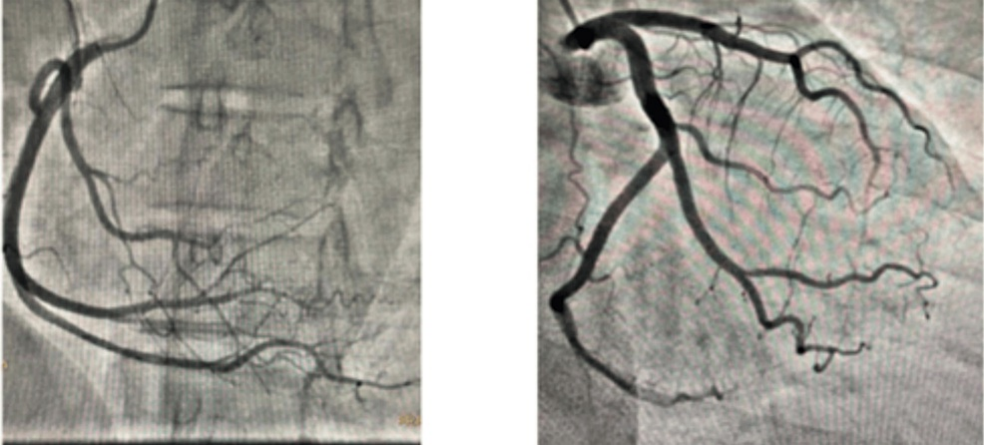
Coronary angiogram The left panel shows normal right coronary (RCA), and the right panel shows normal left coronary artery (LCA)

The patient underwent a treadmill test using the modified Bruce protocol. The electrocardiogram (ECG) at the beginning of the exercise showed a few multiform ventricular premature complexes (VPCs) (Figure 1). Three minutes into the exercise, the heart rate increased to 106 beats per minute, and there was a sudden onset of polymorphic ventricular tachycardia (PMVT) (Figure 2). The exercise test was stopped prematurely, and the arrhythmia subsided within 30 seconds. A coronary angiogram (CAG) was performed to rule out ischemic causes, although she had no coronary risk factors, and it showed normal coronary arteries (Figure 3).

Based on the clinical history and exercise test, there was a high suspicion of catecholaminergic polymorphic ventricular tachycardia, and oral propranolol was started at a dose of 20 mg. thrice daily and gradually increased to 40 mg thrice daily. A sample for genetic testing was sent, which came out to be positive for a mutation in exon coding for RYR2, known to be pathogenic for CPVT. She was advised to avoid participating in competitive sports and any physically strenuous activity. She was also educated about the risk of emotional stress and sudden, startling events. Additionally, her first and second-degree family members were counseled for cascade genetic screening to identify those at risk. Fortunately, her symptoms disappeared in a few days, and 72-hour Holter monitoring during follow-ups at three and six months did not show any abnormalities. The possible requirement of an implantable cardioverter defibrillator (ICD) and/or surgical cardiac sympathectomy denervation (CSD) in the future was explained to the patient in case of recurrence of symptoms despite medical treatment or development of cardiac arrest.

## Discussion

Exercise-induced ventricular tachycardia represents a spectrum of abnormal heart rhythms originating from the heart's ventricles during or after exercise. It may present as palpitations, pre-syncope, syncope, angina, heart failure, or sudden cardiac death. It may occur in patients with or without structural heart disease. Arrhythmia mechanisms include increased exercise workload and oxygen demand, leading to myocardial ischemia. Myocardial ischemia may then induce arrhythmia. Exercise increases sympathetic activity and decreases vagal output, potentially causing arrhythmia [2]. Arrhythmogenic effects of exercise include shortening of the ventricular effective refractory period, increased velocity of impulse conduction, and increased amplitude of afterpotentials [2]. Beta-adrenergic stimulation leads to an increase in the level of intracellular cyclic adenosine monophosphate (cAMP). This, in turn, activates various ion channels, including voltage-dependent calcium currents, transient inward and outward potassium currents, and delayed rectifier potassium currents. On the other hand, α-adrenergic stimulation causes an increase in cytosolic calcium ions. The changes in membrane currents and cytosolic calcium lead to altered action potential duration and refractoriness. This alteration can cause re-entry initiation, increased automaticity, and triggered activity [2]. Exercise-induced ventricular tachycardia may be monomorphic, polymorphic, or bidirectional. Various health conditions, including CPVT, Anderson-Tawil syndrome, MVP, digitalis toxicity, myocarditis, and acute coronary syndrome, can cause exercise-induced polymorphic VT [2]. The first two are inherited channelopathies that affect individuals with a structurally normal heart.

Catecholaminergic polymorphic ventricular tachycardia (CPVT) is a hereditary channelopathy characterized by exercise- or emotion-induced ventricular arrhythmia in structurally normal hearts. It is highly malignant and is an essential cause of syncope and sudden cardiac death in adolescent and adult patients with structurally normal hearts. It is also a significant cause of sudden infant death syndrome (SIDS) [3]. The mean age of onset is 7 to 9 years, but it can also present later in life. The incidence is 1 in 10,000 people. It has a high mortality rate of 31% if untreated [4]. CPVT is classified based on the genetic mutation associated with it. CPVT type 1 (CPVT1) is autosomal dominant with high penetrance and is caused by mutations in the RYR2 (Rhynodine receptor) gene. This gene encodes the cardiac ryanodine receptor responsible for calcium release from the sarcoplasmic reticulum (SR) in cardiac cells. It is the most common form of CPVT, accounting for 50-65% of all cases. Mutations in the CASQ2 gene cause CPVT Type 2 (CPVT2). This gene encodes the cardiac calsequestrin protein, a calcium-binding protein in the SR. Calsequestrin functions as a calcium buffer, and its malfunction can lead to inappropriate calcium release and arrhythmias. CPVT2 is an autosomal recessive, less common, and milder form than CPVT1 [5]. Mutations in other genes like CALM1, CALM2, CALM3, and TRDN have also been linked to the disorder but are less common. During phase 2 of the action potential, calcium enters the myocyte through the L-type Ca channel in the cell membrane. This calcium causes further release of calcium from the SR through RYR2. During diastole, calcium from the cytoplasm re-enters the SR via a calcium ATPase pump or the exterior via a sodium-calcium exchange pump.

Calsequestrin is a calcium-regulatory protein that helps to hold calcium inside the SR during diastole. Mutations in the ryanodine receptor and calsequestrin cause an abnormal leak of calcium from the SR to the cytoplasm during diastole. This excess calcium in the cytoplasm in diastole causes triggered activity by being delayed after depolarization. Exercise and stress further increase the triggered activity by increasing calcium entry into the cytoplasm by cAMP due to catecholamine [5]. CPVT commonly presents as exercise, emotional stress-induced syncope, or sudden cardiac death. Often, the syncope is accompanied by seizure-like activity. Hence, many patients may initially be mistaken for having a neurological disorder. Family history is present in only 30% of patients [4]. ECG is usually normal during rest. Sinus node dysfunction has been noted to occur in patients with CPVT. Sinus bradycardia has been reported to occur in 19% and SVT in 16% of the patients with mutations in RyR2 [5]. The gold standard for diagnosis is reproducible ventricular arrhythmia during exercise testing. Although bidirectional VT is the prototype arrhythmia in CPVT, ill-sustained monomorphic and polymorphic VT can be seen during the exercise test. The ventricular arrhythmias often become more pronounced as the sinus rate increases with exercise into the 110-130 beats per minute range, usually starting as premature ventricular complexes, progressing to ventricular couplets and triplets (often non-uniform QRS morphologies), and degrading into ventricular tachycardia. Epinephrine infusion has been shown to induce arrhythmia and has a sensitivity of 28% and a specificity of 98%. Genetic testing is vital in confirming the diagnosis and identifying at-risk family members. Once a pathogenic mutation is identified in a proband, at-risk family members can be screened for the same mutation (cascade genetic screening). This helps in the early identification and management of affected relatives. Oral beta-blockers are the first line of management. Implantable cardioverter defibrillator (ICD) implantation and surgical sympathectomy are reserved for relapse and refractory cases. Avoidance of stressful situations and competitive sports is an integral part of the management of CPVT.

Andersen Tawil syndrome (long QT syndrome type 7) is a rare inherited channelopathy with autosomal dominant inheritance caused by a mutation of the gene KCNJ2, which encodes the inward rectifier K+ channel Kir2.1. This channel is responsible for the terminal part of repolarization. This syndrome is characterized by a triad of skeletal muscle abnormalities (periodic paralysis), cardiac abnormalities (borderline or mildly elevated QT, prominent U wave in ECG), and skeletal abnormalities (low set ear, ocular hypertelorism, small mandible, clinodactyly in hand and syndactyly in toes, persistent primary dentition, crowded teeth, and multiple missing teeth) [6]. About 60% of affected individuals express complete triads, and 80% express two of the three classic triads [6]. Baseline ECG: mild QTc prolongation, prominent U wave, marked prolongation of the QUc interval. Exercise-induced arrhythmia is seen in a small percentage of affected individuals. They may present as frequent VPC, bigeminy, polymorphic, or bidirectional VT. VT is typically non-sustained, so the risk of sudden cardiac death is rare. Like CPVT, the mechanism of arrhythmia is triggered activity due to delayed after-depolarization caused by an abnormality in calcium homeostasis.

Almost all types of arrhythmias may occur in digitalis toxicity. There are few case reports of exercise-induced polymorphic VT in digitalis toxicity [5]. The primary mechanism is delayed depolarization (DAD). The toxic concentration of digoxin provokes DAD (delayed after depolarization) by increasing calcium in the cytosol, and if the membrane potential reaches the threshold, it initiates a premature action potential or train of action potential (triggered activity). Catecholamine again facilitates this triggered activity.

Exercise-induced polymorphic VT is also seen in a minority of mitral valve prolapse (MVP) cases. MVP accounts for 7% of sudden cardiac death (SCD) in young people and 13% of SCD in females [7]. Exercise-induced polymorphic VT may occur in acute myocardial infarction. It has also been reported in stable coronary artery disease. So, a coronary angiogram should be done to rule out coronary artery disease in patients with exercise-induced VT who have coronary risk factors. Excise-induced VT very rarely occurs in idiopathic VT; however, it is not life-threatening [8].

## Conclusions

Exercise-induced ventricular tachycardia is a rare condition that can be life-threatening for those with cardiac channelopathies. It is essential to recognize this condition early and manage it promptly to avoid mortality. Patients with this condition and their first- and second-degree relatives should receive genetic counseling for early diagnosis and risk stratification. Avoiding competitive sports and strenuous activities is also recommended to prevent sudden cardiac arrest.
